# How causal information affects decisions

**DOI:** 10.1186/s41235-020-0206-z

**Published:** 2020-02-13

**Authors:** Min Zheng, Jessecae K. Marsh, Jeffrey V. Nickerson, Samantha Kleinberg

**Affiliations:** 1 Computer Science Department, Stevens Institute of Technology, 1 Castle Point on Hudson, Hoboken, NJ, 07030 US; 2 Department of Psychology, Lehigh University, Bethlehem, PA, US; 3 School of Business, Stevens Institute of Technology, 1 Castle Point on Hudson, Hoboken, NJ, 07030 US

**Keywords:** Decision-making, Causality, Bayesian networks

## Abstract

**Background:**

Causality is inherently linked to decision-making, as causes let us better predict the future and intervene to change it by showing which variables have the capacity to affect others. Recent advances in machine learning have made it possible to learn causal models from observational data. While these models have the potential to aid human decisions, it is not yet known whether the output of these algorithms improves decision-making. That is, causal inference methods have been evaluated on their accuracy at uncovering ground truth, but not the utility of such output for human consumption. Simply presenting more information to people may not have the intended effects, particularly when they must combine this information with their existing knowledge and beliefs. While psychological studies have shown that causal models can be used to choose interventions and predict outcomes, that work has not tested structures of the complexity found in machine learning, or how such information is interpreted in the context of existing knowledge.

**Results:**

Through experiments on Amazon Mechanical Turk, we study how people use causal information to make everyday decisions about diet, health, and personal finance. Our first experiment, using decisions about maintaining bodyweight, shows that causal information can actually lead to worse decisions than no information at all. In Experiment 2, we test decisions about diabetes management, where some participants have personal domain experience and others do not. We find that individuals without such experience are aided by causal information, while individuals with experience do worse. Finally, our last two experiments probe how prior experience interacts with causal information. We find that while causal information reduces confidence in individuals with prior experience, it has the opposite effect on those without experience. In Experiment 4 we show that our results are not due to an inability to use causal models, and that they may be due to familiarity with a domain rather than actual knowledge.

**Conclusion:**

While causal inference can potentially lead to more informed decisions, we find that more work is needed to make causal models useful for the types of decisions found in daily life.

## Significance statement

Causality is at the core of decision-making, yet little is known about how well people use causal models to make real-world decisions. Methods to go from data to causes have been introduced in machine learning, statistics, economics, and other areas. These algorithms are evaluated based on how accurately they can recover a causal structure, but it is not clear how such models will interact with what individuals already know. In this work we show that causal models can aid decision-making in unfamiliar situations, yet when individuals have prior experience with a domain, causal models can reduce confidence and lead to less accurate decisions. This work has implications for the development of algorithms to find causes, and the presentation of information. First, extracting increasingly complex and detailed models will not necessarily lead to better decisions. Thus, new methods for evaluating the utility of causes are needed. Second, causal information may need to be tailored to each individual’s experience and beliefs.

## Background

Causal relationships let us robustly predict future events, change the future through individual actions and public policies, and look backward to explain why events such as a person’s heart attack happened. A key goal of finding causes, rather than correlations, is to help humans make better decisions. Many computational methods have been developed to extract causal relationships from large quantities of data, for example, to find graphical models of causal links in datasets (Pearl 2000), to uncover the timing of relationships (Friedman et al. 1998), and to identify logically complex relationships (Kleinberg 2012). Given the increasing availability of data, computational methods for extracting causal structure have been applied to a wide variety of problems, including identifying risk factors for heart failure (Kleinberg and Elhadad 2013), finding connectivity from functional magnetic resonance imaging data (Friston et al. 2003), and uncovering causes of sentiment change in online social networks (Bui et al. 2016). While methods for causal inference from data are routinely evaluated on their ability to correctly, completely, and efficiently identify the underlying causal model of a system, the utility of such models in helping people understand real-life decision-making situations has not yet been explored. It is now possible to uncover increasingly more complex and detailed causal models, but it is not known whether these models are necessarily the most useful ones. Thus, there is a need to understand how and when causal models can help people to make decisions.

Prior work in psychology has focused primarily on understanding how people learn about causal structures, rather than on how people use these models for real-world decision-making. Bayesian networks are a common output for machine learning, and many of the models of human causal reasoning are based on causal Bayesian networks (CBNs) (Tenenbaum et al. 2011; Griffiths and Tenenbaum 2005; Rottman and Hastie 2014). There is evidence that the process by which people learn about causes can be captured to a large extent by CBNs, though there are cases where judgment deviates from what would be predicted by a CBN (Rottman and Hastie 2016). Prior knowledge can also affect how we interpret correlations or data (Fugelsang and Thompson 2003; Griffiths et al. 2011), which may have implications for how people combine their knowledge with a CBN to make a decision. However, regardless of whether CBNs can model the process of learning, it is an open question as to whether and to what extent causal information, such as that presented in a CBN, influences decisions.

In particular, prior work has not examined the use of high-level machine learning output (e.g., relationships between variables such as carbohydrates and blood glucose) to make specific choices in daily life (e.g., deciding between items on a restaurant menu). Instead, work on causal learning mainly focuses on testing scenarios that can be understood without prior knowledge.^1^ Such work has focused on making decisions within a model (e.g., which variable to intervene upon to make another variable true), as opposed to how people link newly presented information to what they already know to make real-world decisions. For example, an individual could use guidance on preventing type 2 diabetes to influence their food choices, but this guidance will exist alongside what they already know about diet and health plus their experiences and preferences. Making decisions in a novel system can demonstrate that people understand conceptually how causes work, but this ability does not necessarily imply that people can translate this knowledge to decisions in familiar domains.

Our primary goal in this paper is to understand how causal information can potentially assist people in making decisions, such as what to eat and how to plan for retirement, by better linking actions to goals. While it is not yet known whether provided causal models can actually improve human decision-making, they could potentially reduce cognitive load by acting as heuristics that simplify decisions (Garcia-Retamero and Hoffrage 2006) or by helping to overcome limits on working memory (Easterday et al. 2009). Similarly, causal information could aid decision-making by providing supporting reasons for a choice (Shafir et al. 1993), clarifying the valuation of options (Usher and McClelland 2001; Busemeyer and Townsend 1993), or serving as the basis of task information feedback (Karelaia and Hogarth 2008; Balzer et al. 1992). Influence diagrams, which are visually similar to Bayesian networks, have been used to support communication and decision-making around risk (Fischhoff and Downs 1997; Fischhoff and Davis 2014). Causes are often thought of as intervention strategies (Woodward 2003), and Hagmayer and Sloman (Sloman and Hagmayer 2006; Hagmayer and Sloman 2009) proposed that decisions can be viewed as interventions as well. Understanding the cause of a phenomenon may further determine whether people think an intervention is required at all. For example, Kim and LoSavio (2009) found that when participants were given causal explanations of an individual’s psychological symptoms, they perceived the individual as less in need of treatment compared to when they did not receive such explanations. Despite all of these possible ways that causal information could support decision-making, the hypothesis that causal information can aid in real-world decision-making has not yet been tested.

Furthermore, a core open problem is understanding the interaction between information provided during a decision and an individual’s existing knowledge. In domains where individuals have no personal experience or which they perceive as complex (e.g., decisions about repairing an aircraft engine), causal models may provide welcome aid (Kominsky et al. 2018). However, people are notoriously bad at assessing their own knowledge (Rozenblit and Keil 2002), and therefore they may more often feel they have adequate knowledge without needing the help of a causal model. The interaction between this knowledge and new information (e.g., causal diagrams of varying complexity) is still poorly understood.

For machine learning to have an impact on real-world decisions, we need to better understand how people reason with causes and how this interacts with their prior knowledge and experience. In this work we specifically aim to understand how people use causal information to make the types of decisions found in daily life, rather than decisions that do not relate to prior knowledge and expectations. Understanding this type of decision-making will help us better comprehend how computational methods may actually help the average person. We further aim to understand how the use of causal information is aided or impeded by an individual’s personal experience within a domain, by an individual’s perceptions of their knowledge relative to others, and by an individual’s actual knowledge. In particular, we examine the use of causal information presented as text and as graphical models, as these are the most common output of computational methods for causal inference. Using large-scale experiments on the Amazon Mechanical Turk (MTurk) platform, we test, in a domain where people presumably should have some personal experience (weight management), if additional causal information leads to better decisions (Experiment 1). In Experiment 2, we expand to a domain where people vary more in their familiarity (type 2 diabetes), allowing us to test people with and without personal experience making decisions in this domain. This personal domain experience means they may have existing beliefs and knowledge about the topic, though participants will not necessarily have made decisions in the specific contexts posed in each of our study questions. To further understand the role of experience, we explore how information presented at decision time affects confidence in decisions depending on personal experience in the domain (Experiment 3). Finally, we explore whether people’s perceptions of their knowledge or their actual knowledge may drive our effects (Experiment 4). Across these experiments we demonstrate the intricacies of how causal information can influence decision-making.

## Experimental overview

While prior work has provided insight into causal reasoning and decision-making, we do not yet know how useful causal information is for supporting everyday decisions that relate to an individual’s existing knowledge and experience. We build on prior work showing the possibility of using Amazon’s MTurk platform for studies involving assessing the complexity of causal systems (Kominsky et al. 2018) and for behavioral research more generally (Crump et al. 2013).

We conduct a series of four experiments testing (1) whether causal information improves decision-making, (2) whether the impact of causal information differs for people with and without personal domain experience, (3) how causal information at decision time affects decision confidence, and (4) how perceived and actual knowledge affect use of causal information for decision-making. The four experiments have the same basic structure:

- *Introduction* This page contains general information including expected duration, compensation, and qualifications.
- *Instructions* This screen provides instructions on the task and explains the diagrams that appear in some questions. Participants were told they may be shown diagrams that could assist them with the questions.
- *Decision-making question(s)* The core task involves one or more multiple choice decision-making questions, with one shown on each page.
- *Post-task assessment* This section varied across the experimental conditions: survey on helpfulness of diagrams (Experiments 1–2), assessment of confidence in decision (Experiment 3), and assessment of domain knowledge (Experiment 4). Details are provided within the relevant experiments.
- *Demographic survey* We collect the following information from participants: age, sex, country of birth, race and ethnicity, level of education completed, and current participation in education. For Experiments 2–4, we also collect information about personal domain experience. For Experiments 2–4 we ask whether the individual has diabetes (type 1, type 2, or unsure of status) or is a caregiver for a person with diabetes. For Experiment 4 we additionally ask whether the individual participates in a retirement plan and if they have made active choices in other investment types. The specific questions are provided within each relevant experiment.
- *Debrief* Participants are shown a post-task information screen informing them of the purpose of the study.
- *Feedback* Finally, we provide a form for open-ended comments or feedback on the task.

Based on the expected duration (15 min) and minimum wage at the time of the study, we paid $2 for completing the task. Experiment 4 was longer (approximately 20 min), so payment was increased to $2.75. The task was open to all US resident MTurk workers aged 18–64 who could understand written English. Individuals were only able to complete one survey, and they were prohibited from using the back button to ensure questions were answered in the order presented. We did not restrict to workers who have completed a specified number of tasks or who have achieved a certain approval rating. We communicated an estimated task time of 15 min (20 min for Experiment 4), but allowed 60 min. The task was posted during daytime hours on weekdays to reduce the effects of time of day on demographics while allowing us to meet recruiting targets. Using the TurkPrime microbatch feature (Litman et al. 2016), each task was reposted in small batches, so samples were collected evenly throughout the day. For each condition in each experiment, we used a sample size of at least 100. To calculate this sample size, we ran small pilot versions of each experiment to estimate the expected differences in proportions. For each experiment, using the estimated proportion difference and assuming a power of 0.8, we found that approximately 100 participants per condition should be sufficient to detect differences between groups with 95% confidence while also allowing for participant exclusions. Experiment-specific considerations regarding sample size are described within the participants section of each experiment.

## Experiment 1

Our first experiment is designed to test whether the type of causal information extracted by machine learning methods is beneficial for the kinds of decisions made in daily life. That is, rather than only learning how a novel system works or making decisions in scenarios where people have no prior experience, real-world decisions such as what to eat or whether to walk somewhere or drive involve combining prior knowledge and experience with any new information presented. Causal information, and particularly that represented in diagrams, could potentially improve decision-making by reducing cognitive load. For example, work on learning has shown that causal diagrams can improve synthesis and retention of knowledge (Corter et al. 2011), and other work suggested causal information can reduce cognitive load by serving as heuristics (Garcia-Retamero and Hoffrage 2006). We now test whether seeing causal information at the time of decision leads to better decision-making.

### Method

#### Participants

A total of 1800 people recruited through Amazon MTurk participated in the study. There were 18 possible combinations of questions (3 causal information question versions x 3 control question versions x 2 orderings). We were unsure if the ordering or combination of questions may influence results, so we recruited a sample of 100 participants for each combination per our minimum sample size. Participants were US residents, aged 18–64. Participants’ data were excluded if they reported being outside the allowed age range, if they failed to complete the survey, or if they submitted an incorrect code at the end of the survey. Of the 1800 participants, 76 submitted an incorrect code or failed to complete the study, and 6 reported being outside age 18–64. Thus, 1718 participants remained in the analysis. Detailed demographic information for all participants can be found in the Appendix.

#### Materials

For the decision-making task we selected a domain about which we believed a sample of American participants would have ample experience making decisions: weight management. Note that we do not mean that individuals necessarily have experience in the specific decision type posed in the problem, rather that they are likely to have thought about the domain (weight management, making diet and activity choices) and are likely to have beliefs about said domain. Participants were shown a short scenario and asked to give advice to another person. We believe that this set-up is more likely to focus the participants on the question as presented rather than elicit idiosyncratic preferences from their own life (see (Polman 2012) and (Lu et al. 2013), for example). For the causal information, we use a model that is relatively simple but reflects current guidance on factors affecting bodyweight.^2^ As a result, there are multiple direct causes of weight, as well as an indirect cause. The decision-making question used is as follows.

**Bodyweight real-world question:**

Jane just started college and is adjusting to her busy schedule of classes and extracurricular activities. She has heard about the “freshman 15,” where new college students gain 15 pounds during their first year of college. Jane wants to avoid this, while also having fun, making new friends, and leaving time for homework and studying.

What is the ONE thing you think Jane should do to achieve her goal?

- A. Go for a 30-min walk every weekend
- B. Maintain a healthy diet
- C. Avoid hanging out with friends
- D. Watch less TV

Answer choices were presented to all participants in the order shown. The correct answer was designated as the choice that was the most direct cause of weight change, i.e., choice B. Detailed explanations for this and other answers can be found in the Appendix.

**Fig. 1:**
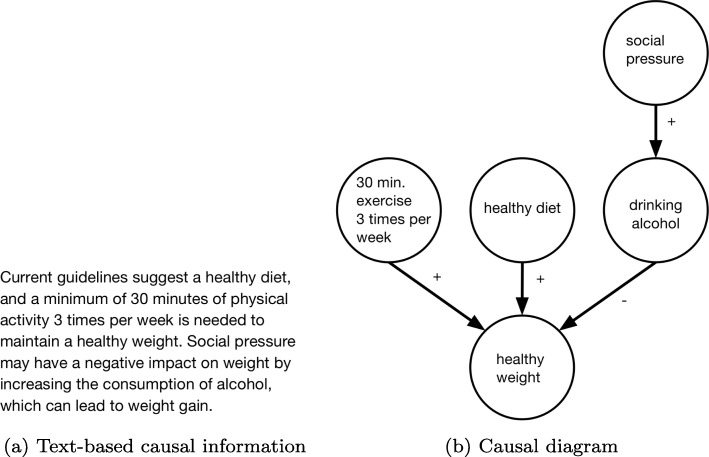
Causal information used in (**a**) text and (**b**) diagram conditions of Experiment 1

To augment the decision-making question, we created two different formats of causal model information that are relevant to the problem and reflect the type of guidance and level of detail commonly provided to individuals. We created a causal text addition that described current guidelines for how to manage bodyweight (Fig. 1a). We also created a causal diagram, seen in Fig. 1b, that presented the same information as the text in a graphical format akin to a CBN. We augmented edges (i.e., the arrows) with “+” or “–” signs to indicate whether the cause produces or prevents the effect.

#### Procedure

Participants were randomly assigned to receive one of three information conditions in answering the real-world question: (1) no extra information beyond the question (no info condition; *n*=573), (2) text-based causal information (causal text condition; *n*=572; see Fig. 1a), or (3) a simple causal diagram (causal diagram condition; *n*=573; see Fig. 1b). For the causal text and causal diagram conditions, the extra information was displayed visually in between the question and the answers. In the instructions for the task, participants were informed that some questions may have diagrams to assist them, and they were provided with an introduction to the meaning of the diagrams as well as the meaning of features such as plus or minus signs along the edges. Participants who were shown a diagram received a questionnaire afterward asking: *Did you consult the table or diagram when answering the FIRST/SECOND question? This is the question that asked about weight management.* Answer choices are: Yes, and it was helpful; Yes, but it did not affect my answer; No; Not sure/can’t remember.^3^

Participants also received a question that did not pertain to real-world knowledge, namely a question about a blicket detector (*n*=1718). This question also varied the causal information presented to aid with decision-making. The version of this question that included a causal diagram may have been confusing, as it was intended to convey a lack of effect but may have been interpreted by participants as conveying a preventative causal relationship. As such, we do not feel the results of this question can be interpreted meaningfully and so we do not include the results of this question in the analyses here. Participants were randomly assigned to receive the real-world or the control blicket question first. There was no effect on performance on the real-world question regardless of the order in which it was answered (*p*=0.2943). Thus, we collapse across conditions for participant responses from the real-world question regardless of whether it was answered first or second. We revisit the comparison of a control question that did not involve real-world knowledge in Experiment 4.

### Results and discussion

**Effect of causal information on decision-making**Our main question of interest was whether people would be more likely to pick the correct behavior if provided a causal diagram. In the following analyses, we compare the percentages of people who chose the correct answer across conditions. We test for significant differences in these percentages in this experiment and in all subsequent experiments using two-tailed Fisher exact tests and report odds ratios (ORs) to provide insight into effect sizes.

**Fig. 2:**
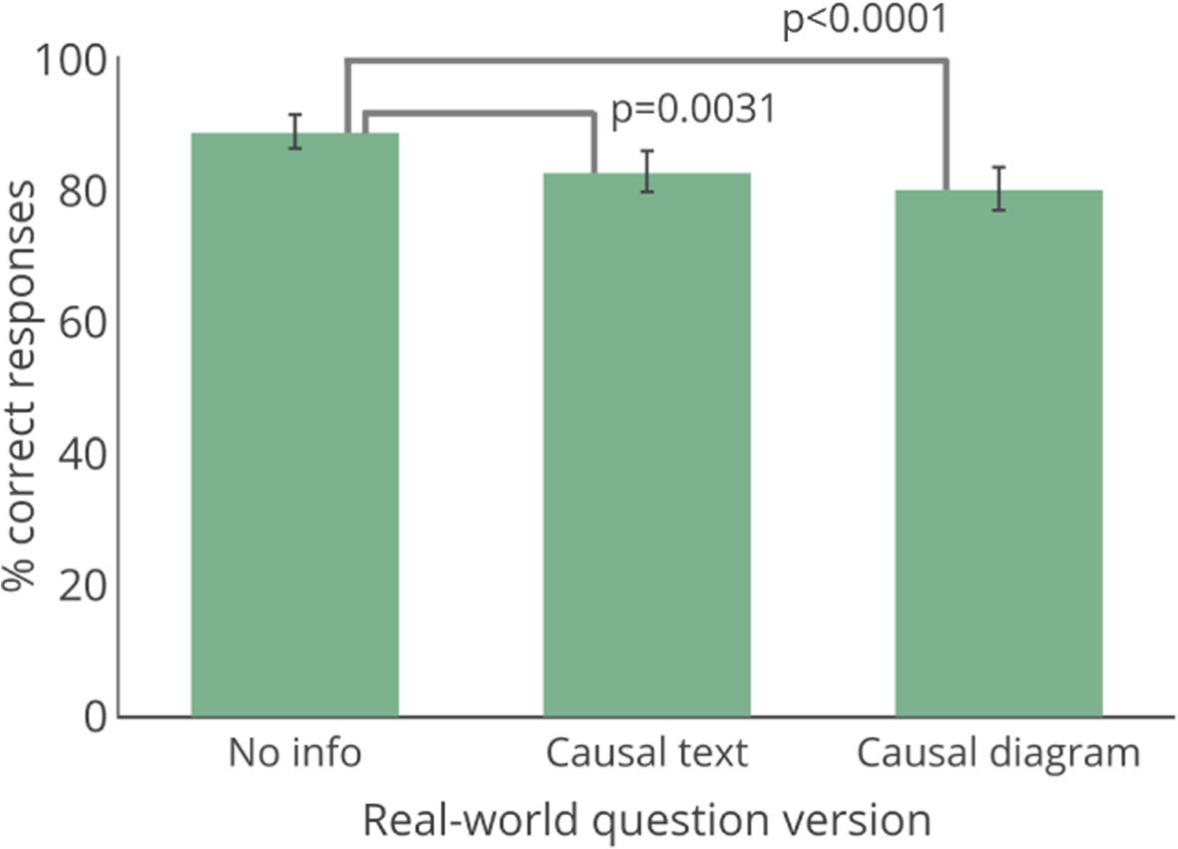
Results for Experiment 1, with response accuracy by condition. Error bars show 95% confidence interval

As shown in Fig. 2 and Table 1, a large percentage of participants (88.8%) picked the correct answer in our decision-making paradigm when no extra causal information was provided.^4^ However, contrary to our expectations, causal information did not lead to better decisions. Fewer participants correctly answered the question when given more information, regardless of whether it was presented as causal text (82.7% correct responses) or as a causal diagram (80.1% correct responses). Thus, a causal diagram led to 8.7% fewer correct responses than no information at all (*p*<0.0001, *OR*=1.98), and causal text led to 6.1% fewer correct responses (*p*=0.0031, *OR*=1.66). The difference between the diagram and text conditions was not significant (*p*=0.2877).^5^ Accuracy in answering the real-world question in the causal diagram condition was not driven by perceived helpfulness of the diagram, in that there was no difference in the percentage of people who correctly answered the question who said the diagram was helpful compared to those who said it was not (helpful and informed answer = 80.7% correct, not helpful = 86.5% correct, *p*=0.4439).^6^

**Table 1:** Percentage of respondents selecting each option across the three conditions in Experiment 1

	A. Walk	B. Diet	C. Friends	D. TV
No info	9.1	88.8	0.9	1.2
Causal text	14.5	82.7	1.9	0.9
Causal diagram	14.5	80.1	4.4	1.0

Answer B is correct

Our findings are surprising given that past work has suggested presenting more information (often in the form of complementary text and diagrams) results in better inferences (Mayer 2014). One possible explanation of our results is that people are doing worse when shown causal information (regardless of whether it is represented as causal text or a causal diagram) precisely because they have experience in this decision-making domain. This may be related to the expertise reversal effect, which posits that the more someone knows, the less useful some enhancements such as visualizations are (Kalyuga et al. 2003). We test this possibility in Experiment 2.

## Experiment 2

In Experiment 1 we showed how causal information, presented as either text or a diagram, can lead to fewer correct responses in real-world decisions where information must be combined with existing knowledge and transferred from these general causal claims to specific options. In that experiment we used a scenario where most people can be expected to have prior knowledge or beliefs. We now follow up on this using a decision-making scenario where we can expect some participants to be less experienced than others. While people with prior beliefs may experience conflicts between the diagrams and their existing understanding, people without experience in a domain may be more likely to take the new information at face value.

To test this, we employed the example of managing type 2 diabetes (T2D), using diet and exercise to keep blood glucose (BG) in a healthy range. We chose this question as diabetes affects a substantial portion of the US population (so we can expect a sample of MTurk workers to include people with diabetes) and it is challenging to manage, as many other factors also affect glucose, including stress and physical activity. If personal domain experience is truly responsible for lower accuracy with the causal diagram, then participants with diabetes experience should perform closer to the sample of Experiment 1, while inexperienced participants should perform better than experienced participants in the causal diagram condition. While the question relates to diet and exercise (as these are important for managing T2D), this is in the context of managing diabetes, so we believe that most participants without experience managing diabetes will have few beliefs about the causal structure.

### Method

#### Participants

A total of 600 people recruited through Amazon MTurk participated in the study and were compensated $2.00. Participants were US residents, aged 18–64. We used a sample of 300 individuals per condition to recruit a sufficient number of individuals with diabetes experience, while still representing a random sample of MTurk workers. We did not mention diabetes in any way in the question description or qualifications, to ensure a broad sample of the population and to also ensure that participants did not think diabetes status was a requirement of the experiment (something that could have interfered with their truthful answering of the experience question). Of these participants, 7 failed to complete the study, and 3 were outside the age range, leaving 590 participants for analysis. Of the participants meeting the inclusion criteria and completing the survey, 23 had T2D, while 46 did not have T2D but were caregivers for someone with diabetes. We did not specify what it means to be a caregiver, and thus can expect this group to include spouses, parents of children with diabetes, and children of individuals with diabetes. We excluded the 8 individuals who reported they had type 1 diabetes or were unsure of their exact diagnosis in our analysis, due to the small size of the groups (582 participants remained in the analysis).

#### Materials

The decision-making question in the domain of diabetes was constructed to have low complexity, while reflecting common guidance on managing blood sugar without insulin.^7^ That is, since this is a less familiar setting to most participants than the one in Experiment 1, we employed a simpler model than the one in that experiment to avoid confounding due to any effects of complexity. The diabetes question was as follows.

**Diabetes real-world question:**

Bob was recently diagnosed with T2D. His body does not produce enough insulin, so after a meal, his blood sugar may become dangerously high. Bob does not want to inject insulin, and was relieved when the doctor said his diabetes could be controlled with diet and exercise and ensuring he maintains a healthy weight.

Bob has had a stressful week at work and is looking forward to seeing his friends Friday evening. They usually meet at Bob’s favorite fast food restaurant for hamburgers, but now he wonders if that’s okay.

What is the BEST suggestion you can give Bob to keep his diabetes under control and avoid needing insulin injections?

- A. Walk to dinner
- B. Order a grilled chicken sandwich instead of a hamburger
- C. Order a grilled chicken salad and ask his friends to go for a bike ride
- D. Do what he usually does

The correct answer here is C, as the question asks for the best suggestion and this one addresses two causes. Note that the question set-up used in all conditions includes general information about diabetes management, but does not put it in causal terms. Answer choices were presented to all participants in the order shown.

#### Procedure

**Fig. 3:**
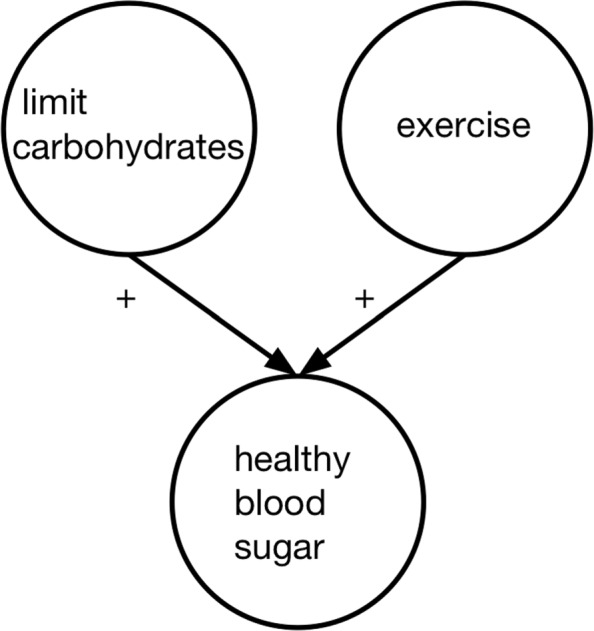
Experiment 2 causal diagram

The same basic procedure was used as in Experiment 1 with the following changes. Participants were randomly assigned to one of two information presentation conditions: (1) question only (no info condition; *n*=289) or (2) question augmented with a simple causal diagram (causal diagram condition; *n*=293) (Fig. 3).^8^ We did not include a causal text condition in this experiment because the results of Experiment 1 suggested it did not differ from the causal diagram condition. After answering the diabetes decision-making question, participants answered the same demographics questions as in Experiment 1. In addition, they were asked “Have you ever been diagnosed with diabetes?” Response options were: Yes, Type 1; Yes, Type 2; Yes, unsure which type; No, but I am a caregiver for someone with diabetes; and No. This was done to allow comparison of results between those with experience managing diabetes and those without such personal experience.

### Results and discussion

**Fig. 4:**
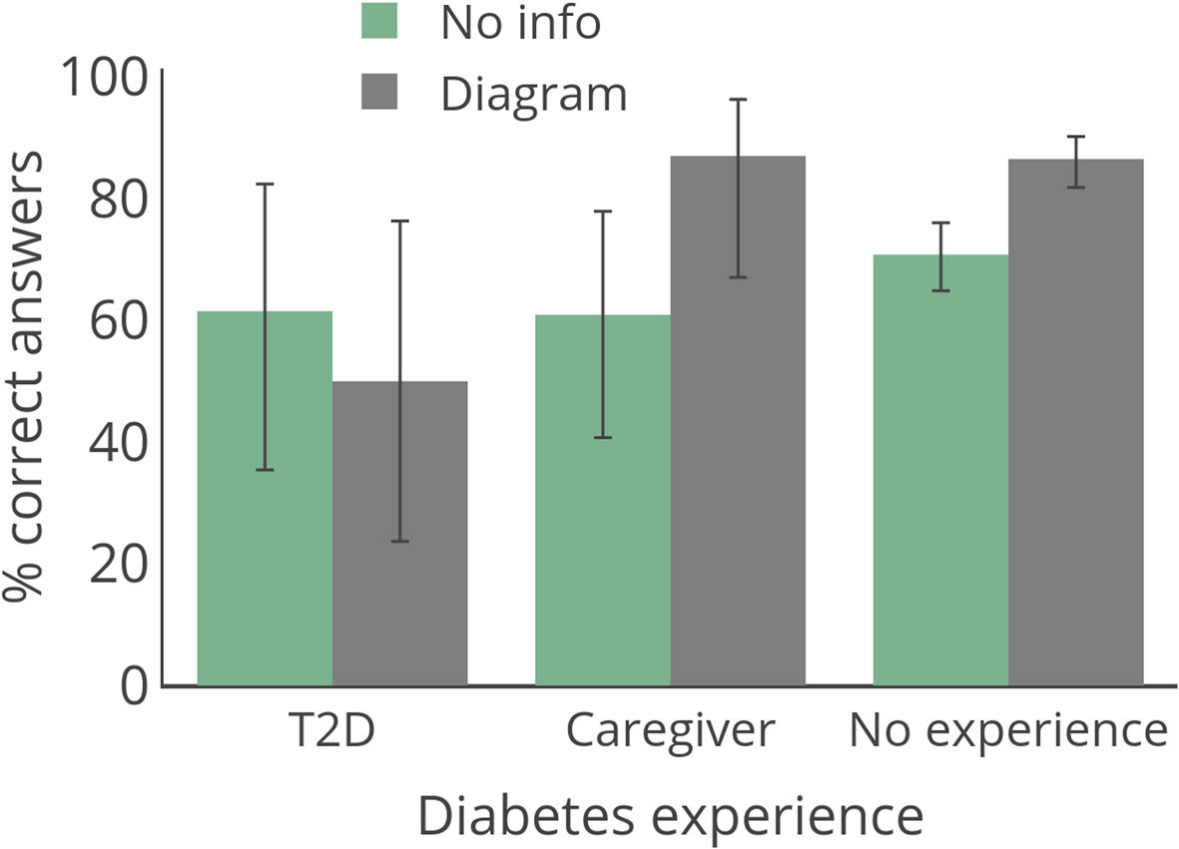
Results for Experiment 2. Error bars show 95% confidence interval

**Effect of personal domain experience on decision accuracy with causal information** Figure 4 and Table 2 show the percentage of participants who correctly answered the decision-making question for Experiment 2 separated by the three personal domain experience groups of T2D, caregiver, and people without diabetes (PWOD) for each of the two information conditions (no information vs. causal diagram). We separated participants who reported being a caregiver because it was unclear if that indirect form of experience would change how causal information was used. Inspecting the means shown in Fig. 4 suggests the caregiver and PWOD groups have similar patterns of responses across the two information conditions. Statistical comparisons supported this conclusion.^9^ To simplify further comparisons, we collapse these two groups as one no personal experience group.

**Table 2:** Percentage of respondents choosing each answer in Experiment 2, broken down by level of diabetes experience

Condition	Subgroup (*N*)	A. Walk	B. Sandwich	C. Salad+Bike	D. Usual
No info	T2D (13)	7.7	30.8	61.5	0.0
Causal diagram	T2D (10)	20.0	30.0	50.0	0.0
No info	Caregiver (23)	13.0	26.1	60.9	0.0
Causal diagram	Caregiver (23)	4.3	8.7	87.0	0.0
No info	No experience (253)	6.3	20.6	70.8	2.4
Causal diagram	No experience (260)	2.7	9.2	86.5	1.5

Answer C is correct. *N* is the number of individuals in the subgroup

Our major question of interest is whether performance in the information conditions differs as a function of personal experience. Overall, our results suggest that additional causal information may be useful to people without personal experience in a domain, but not to people with such personal experience. To test this interaction question, we conducted a two-step binary logistic regression with accuracy for the decision-making question (incorrect vs. correct) as our outcome. We entered information condition (no info vs. causal diagram) and personal domain experience (T2D vs. no personal experience) as variables in the first step of the model. The interaction of these two variables was entered in the second step. The final model was significant, with *χ*^2^(3)=28.76,*p*<0.001, Nagelkerke *R*^2^=0.074. In Step 1, information condition (*OR*=2.534,*p*<0.001) and personal domain experience (*OR*=2.726,*p*=0.024) were significant predictors. These effects should be interpreted in the light of a marginally significant interaction in Step 2 (*OR*=4.436,*p*=0.090).

To explore the interaction between personal domain experience and information condition, we used two-tailed Fisher exact tests for specific comparisons and reported *p* values after Benjamini-Hochberg correction. We found that in the no info condition, which only describes the individual’s doctor’s recommendation, the group with no diabetes experience had 69.9% correct responses. There was not a statistically significant difference between the no experience group and the T2D group (61.5%, *p*=0.6850). In contrast, when provided with a simple causal diagram, the T2D group had only 50.0% accuracy, while the no experience group performed significantly better (no experience = 86.6%; *p*=0.0156;*OR*=6.45). The higher percentage of people who correctly answered the question comparing no info to causal diagram for the no experience group was significant, with a substantial increase in correct responses (*p*=0.0004,*OR*=2.77). The decrease in accuracy for people with T2D was not significant (*p*=0.6850,*OR*=0.63).

It is notable that the no personal experience group had a higher rate of correct responses when shown the causal diagram (87% vs. 70%), an opposite pattern to Experiment 1 where the percent of correct responses was higher with no info (89%) than with a causal diagram (80%). Thus, it does not appear to be the case that in general people are unable to use causal diagrams to make decisions, or that there is something inherent in causal information that leads to bad decisions. Instead there seems to be an interaction between a person’s prior domain experience and the effect of causal information on decision-making. This experiment provides further support for our hypothesis that personal experience in a domain leads to challenges in using causal information during decision-making. One possible explanation is that individuals with domain experience may have their own mental representation of a phenomenon and may struggle to integrate the diagrams we show them with this mental representation. That is, people with experience in a domain may have high belief in their knowledge of that domain, but this confidence may reduce their ability to make use of new information. We test these possibilities in Experiment 3.

## Experiment 3

One explanation for the results of our first two experiments is that new causal information may conflict with existing causal models and prior beliefs. For people with personal experience in a domain, receiving information that challenges their held beliefs may reduce confidence in their understanding. For people without experience and no strongly held beliefs about the causal workings of a domain, receiving causal information may be a welcome addition that aids decision-making. In Experiment 3 we test this possibility by using the question from Experiment 1 (to examine confidence in a case where most people have experience) and from Experiment 2 (for a case where most people do not have experience).

### Method

#### Participants and procedure

We re-ran Experiments 1 and 2 with 1000 new individuals. The study design followed the same procedure as in Experiments 1 and 2. Participants completed one real-world decision-making question, either diabetes or weight. The key difference is that, after the decision-making question, we added a question asking: “How confident are you in your answer to the previous question (about diabetes/weight)?” Answer choices were: very, somewhat, not at all.

As in the previous experiments, we excluded participants who did not complete the study or were outside the age range of 18–64 (*n*=54). We used a sample of 200 participants per condition, with the goal of striking a balance of recruiting sufficient numbers of participants with diabetes experience for the diabetes question, while using the same sample size for the weight conditions and not overstressing the MTurk pool. In our sample, there were too few participants with diabetes experience (6 type 1, 15 type 2, 2 unsure what type, and 36 caregivers) to analyze these subgroups across the different question versions, so we focused on people without diabetes (no experience) in the analysis of the diabetes question. Of the 1000 participants (600 for weight’s three versions: no info, causal text, causal diagram; 400 for diabetes’s two versions: no info, causal diagram), we are left with a total of 887 participants for analysis (567 for weight and 320 for diabetes).

### Results and discussion

**Replicating previous findings** First, we check to see if the basic effects of the previous experiments replicate in this sample. As seen in Experiment 1, fewer participants correctly answered the question on weight when provided with additional causal information, as seen by comparing the no info condition (89.1% correct) to the causal diagram condition (77.4% correct; *p*=0.0024,*OR*=2.40) and the causal text condition (81.3% correct; *p*=0.0421,*OR*=1.88). As in Experiment 2, for the diabetes question significantly more participants without diabetes chose the correct answer when using the causal diagram (86.6%) than in the no info condition (66.9%; *p*<0.0001,*OR*=3.21). While this was not the goal of this experiment, it provides evidence that the effect is stable.

**Interaction between prior experience and causal information for decision-making confidence** Our primary hypothesis in this experiment was that participants who had experience in a domain would grow less confident after seeing new information, while those without domain experience would become more confident. Table 3 shows results for both the weight and diabetes questions, where we observe exactly this effect. To test this interaction prediction, we conducted a logistic regression on confidence judgments. Since only 6 total participants reported being not at all confident, a multinomial regression across all three confidence levels was not viable. We excluded par- ticipants who reported being not at all confident from further analyses. To keep the questions identical to those for Experiments 1 and 2, both the diabetes and bodyweight questions included no info and causal diagram conditions. The bodyweight question further had a causal text condition, as in Experiment 1. To align our statistical comparisons across the two domain conditions, we excluded the causal text condition for the bodyweight question from the following logistic regression. We conducted a two-step binary logistic regression with confidence for the decision-making question (very confident vs. somewhat confident) as our outcome. We entered information condition (no info vs. causal diagram) and domain (bodyweight vs. diabetes) as variables in the first step of the model. We entered the interaction of information condition and domain in the second step. The final model was significant, *χ*^2^(4)=102.66,*p*<0.001, Nagelkerke *R*^2^=0.192. In Step 1, domain was a significant predictor (*OR*=0.403,*p*<.001). This effect should be interpreted in the light of a significant interaction in Step 2 (*OR*=0.232,*p*<0.001).

**Table 3:** Participant self assessment of confidence in Experiment 3

	Weight management confidence			Diabetes management confidence (no experience)		
	Very	Somewhat	Not at all	Very	Somewhat	Not at all
No info	82.6 (91.5)	17.4 (78.1)	–	41.7 (86.8)	54.8 (52.8)	2.5 (50.0)
Causal text	73.6* (83.8*)	26.0* (76.0)	0.4 (0.0)	–	–	–
Causal diagram	69.5** (81.8**)	30.0** (66.7)	0.5 (1.0) ^*†*^	68.8*** (93.5)	31.2*** (71.4*)	–

Percentage of correct responses for participants reporting each level of confidence is shown in parentheses. Statistical significance for comparisons against the no information condition, using *p* values after Benjamini-Hochberg correction, are indicated by * for *p*<0.1, ** for *p*<0.05, and *** for *p*<0.005. Some edge cases are indicated: ^*†*^*N*=1, – indicates no data in that cell

To explore the interaction between domain and information condition, we used two-tailed Fisher exact tests with Benjamini-Hochberg corrections to test specific comparisons. As predicted under our hypothesis, fewer participants in the bodyweight condition (where we expect most people to have some prior experience or beliefs) are very confident in their answers with causal diagrams relative to no information (no info = 82.6% very confident, causal diagram = 69.5% very confident, *p*=0.005, *OR*=2.09). For the diabetes question, where we analyze data only from participants with no experience, we find the opposite effect from that of the weight question, as predicted. Here the causal information leads to more no experience participants being both very confident and accurate. We find that while 41.7% of individuals with no experience with diabetes were very confident in their answers in the no info condition, 68.8% of participants with no experience reported being very confident in the causal diagram condition (*p*<0.001,*OR*=3.08). In contrast to the weight condition, where subjects were less confident and less accurate when shown more information, here causal information led to both a higher percentage of correct answers and higher confidence. Together these results suggest that the challenge is not in using the diagram itself (as people with no prior experience are in fact aided by it), but rather in integrating the diagram with prior knowledge.

We note that across questions and conditions, as shown in Table 3, the groups who were more confident in their answers did perform better on average than those who were less confident (comparing accuracy among people rating themselves as very vs. somewhat confident, on diabetes: *p*=0.0030,*OR*=5.77 for causal diagram, *p*=0.0013,*OR*=5.87 for no info; on weight: *p*=0.0809,*OR*=2.25 for causal diagram, *p*=0.0823,*OR*=2.99 for no info). However, we solicited confidence after participants answered the question, which may provide insight into a potential mechanism. The illusion of explanatory depth suggests people believe they understand how things work much better than they actually do (Rozenblit and Keil 2002). While people initially overestimate their understanding, they significantly lower their self-estimates of their knowledge after being asked to explain phenomena such as how a zipper works. Relevant to our study, the illusion has also been found in the health domain, specifically in understanding mental disorders (Zeveney and Marsh 2016). Focusing specifically on the causal connections of a process can help mitigate overestimation by identifying knowledge gaps (Johnson et al. 2016). Our two questions provide insight into cases where most people have decision-making experience (weight) and where most do not (diabetes). Answering the decision-making question may have revealed to people how much or how little they know. In Experiment 4 we build on this by specifically exploring perceived and actual knowledge.

## Experiment 4

The first three experiments in this paper found that causal information at the time of decision can lead to lower accuracy for people with domain experience, while aiding those without experience. In addition, we showed that causal information reduces confidence in experienced individuals while increasing it in novices. A limitation of the experiments is that we did not examine actual knowledge in each domain, but rather assumed that most people would have made decisions about weight management before, while most people without diabetes would not have spent time managing diabetes. However, this does not mean that individuals with diabetes experience are managing their diabetes successfully or that those without personal diabetes experience may not have some knowledge or beliefs about it.

We believe there are three key facets of knowledge about a domain that are relevant here: personal domain experience, actual knowledge, and perceived relative knowledge. Personal domain experience is what we provided evidence toward in Experiment 2: whether people have previously made decisions in the domain regardless of whether or not the decisions are correct. Actual knowledge, or proficiency, in contrast, does not require any experience but concerns whether people have accurate knowledge about a domain. Finally, perceived relative knowledge is belief in one’s ability to accurately make decisions in the domain.

In this experiment we now test how these three individual factors affect the use of causal information at decision time. If lack of experience is primarily responsible for the improvement in accuracy in Experiment 2, we expect to see low levels of experience leading to higher accuracy with causal information, and high levels of experience leading to lower accuracy with causal information. As hypothesized in previous experiments, it may be that experience provides people with their own causal models in which they feel confident, and that these models are then hard to update with new causal information. We will use self-perceived knowledge relative to others as a measure of whether people have strong beliefs in their existing causal models. Like experience, we expect that people who rate their own knowledge as high will show lower accuracy when provided with causal information, whereas people who do not rate their own knowledge as high will benefit from causal information. We believe that the effect we observed hinges on perceived knowledge, not actual knowledge. As such, we do not predict that actual knowledge will influence accuracy in the same way.

We further aim to expand our investigation to determine if our findings are specific to the health domain. Specifically, we now include a real-world decision related to finance to determine if the influence of causal information is specific to health. Additionally, we include control questions involving scenarios that are not intended to be related to prior knowledge to evaluate whether participants’ difficulty in using causal information is specific to cases that conflict with previous knowledge of a causal system.

### Method

#### Participants

We recruited 600 MTurk participants, with the goal of a sample of 200 per condition, to be consistent with Experiment 3. Eleven participants failed to complete the study and 3 reported being outside the age range. Of the eligible participants completing the diabetes question, an additional 23 individuals reported a diagnosis of type 1 diabetes (T1D) or were uncertain of what type of diabetes diagnosis they had. These individuals were excluded from analysis of the diabetes question, for consistency with Experiments 2 and 3. As such, of the 586 participants, a total of 563 participants had complete data that were analyzed for the diabetes-related question: 19 individuals with T2D, 41 caregivers, and 503 individuals with no diabetes experience. For the finance question, 326 reported having experience making active choices in retirement plans, 249 reported not having experience, and 11 were unsure of whether they had such experience. To be consistent with our removal of participants who were unsure about their status in previous experiments, we only looked at the experience and no experience participants for the finance question. To obtain a more direct comparison to the finance decision, we analyzed only the no diabetes experience and the T2D groups for the diabetes question (a total of 585 participants remained in the analysis).

#### Materials

For this experiment, we introduce two new real-world decision-making questions (in personal finance and diabetes management) along with three new control questions. For the real-world questions, we used the three information conditions of Experiment 1: (1) no extra information beyond the question text (no info condition), (2) text-based causal information (causal text condition), and (3) causal diagram (causal diagram condition). The control questions were included to test whether individuals are able to use causal information effectively on its own when it is not combined with prior knowledge. Since these questions are about novel domains, there is no “no information” condition, since participants in that case would only be able to guess. Thus, control questions presented causal information in both conditions, either as (1) text-based causal information or (2) a causal diagram. Additionally, we tested three different causal structures in the control questions: a common effect structure with only three relevant nodes (Fig. 6a and b), a more complex structure with the common effect structure embedded (Fig. 6c and d), and a causal chain (Fig. 6e and f).

The five decision-making questions used are as follows. We first introduce the two real-world decision-making questions, and then the three control ones.

**Real-world personal finance question:**

James has been working full-time for the last 30 years and is hoping to retire in the next few years. He currently has his retirement savings invested in a mix of stocks, bonds, and real estate. As he gets closer to retirement, he now wants to reduce the risk in his retirement portfolio, to ensure he does not lose this money in the event of a market crash or another unforeseen event.

Knowing that James’s MAIN goal is reducing his risk, which option would you advise him to choose?

- A. Sell 15% of the stock he owns in the company he works for and buy stock in a different company
- B. Sell 15% of his stock holdings and keep that money in a savings account
- C. He shouldn’t make any changes close to retirement
- D. Use money from his emergency savings account to buy more stocks

**Fig. 5:**
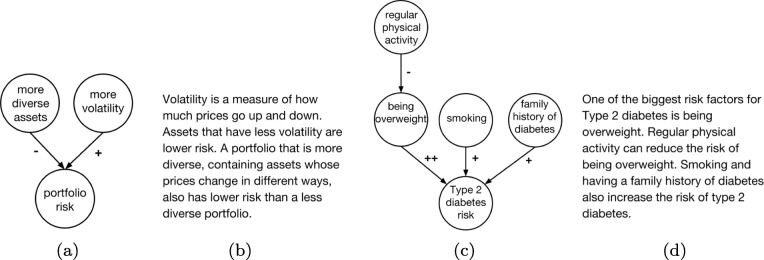
Causal information for real-world questions in Experiment 4. Figures were scaled with nodes at the same size. **a** Finance diagram. **b** Finance text. **c** Diabetes diagram. **d** Diabetes text

The causal text and causal diagrams that augment this question are shown in Fig. 5a and b. For the causal diagram version, we also provided brief text explaining the terms used. The explanation read: “*This figure shows causes of risk. Volatility is a measure of how much prices go up and down, and a diverse portfolio contains assets whose prices change in different ways.*” The correct answer for the question is B, as it is the only one that reduces volatility and increases diversity. The question text is based on introductory guidance given to individuals about investing.^10^

**Real-world diabetes question:**

Alan’s brother was recently diagnosed with T2D, and Alan is now concerned about his own risk of developing diabetes. He works long hours in a high-stress job, which often leaves him too tired to cook healthy meals. Normally he likes to come home from work and relax by watching television while drinking a soda. He occasionally smokes, mostly when he has had a tough day at work.

What would you advise Alan to do if he wants to reduce his risk of T2D?

- A. Focus on helping his brother manage his diabetes
- B. Get takeout on his way home so he does not have to cook
- C. Drink only water when he is at home, and take a 15-min walk to the grocery store when he wants a soda
- D. Reduce his smoking

The text and diagrams that augment this question are shown in Fig. 5c and d. Answer C is the correct choice, as it fulfills the strongest cause according to the text and diagram.

**Fig. 6:**
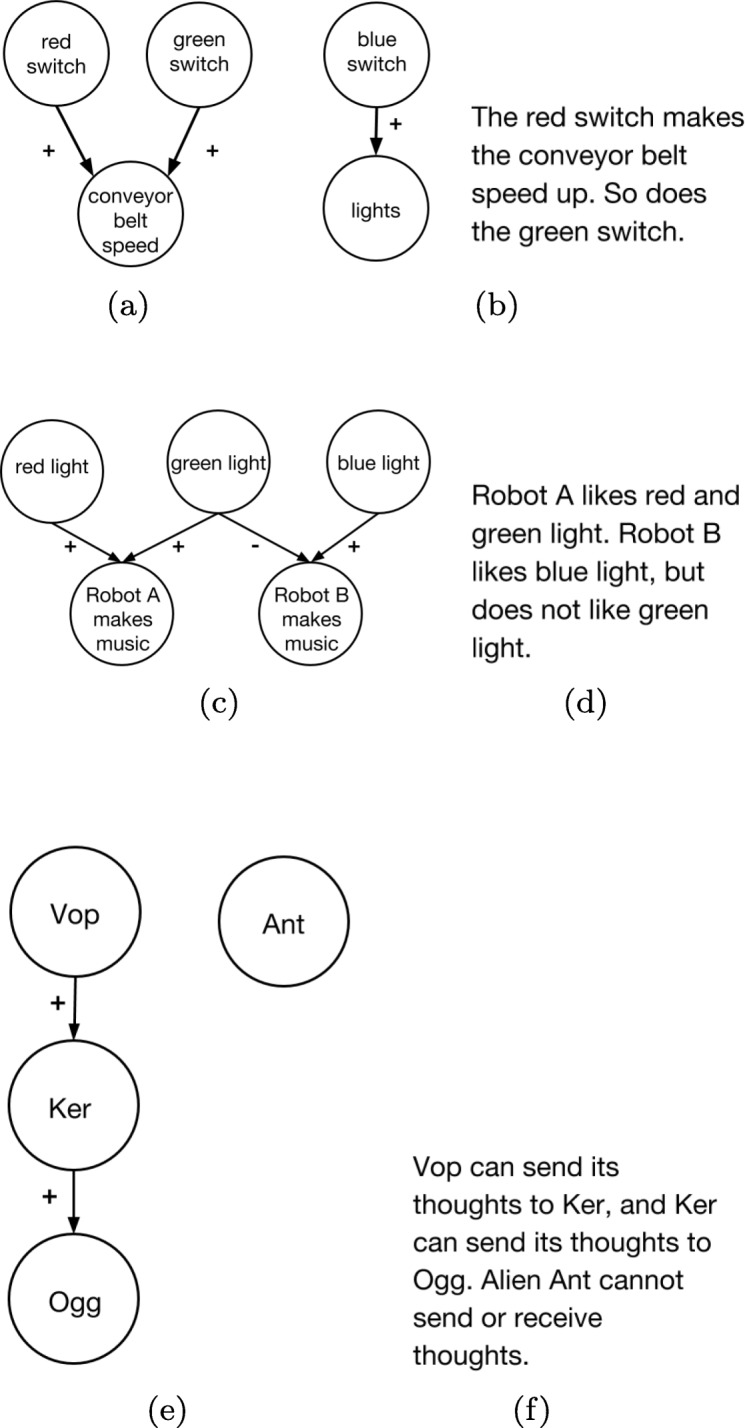
Causal information for control questions in Experiment 4. **a** Common effect diagram. **b** Common effect text. **c** Common effect and common cause diagram. **d** Common effect and common cause text. **e** Causal chain diagram. **f** Causal chain text

We now introduce the three control questions. The names used for these questions refer to the overall causal structure depicted in the description and shown in Fig. 6.

**Control common effect question:**

Tim has a factory that makes widgets. In the factory is a conveyor belt that moves the widgets to boxes so they can be shipped to stores. To control the conveyor belt there are three switches: red, blue, and green. The blue switch turns on the lights above the conveyor belt.

Some of the other switches can change the conveyor belt’s speed. If two switches are on that make the belt speed up, it goes even faster.

Tim wants to make the conveyor belt go as fast as possible. What should he turn on?

- A. Red switch
- B. Green switch
- C. Red switch and green switch
- D. Blue switch

The correct answer is C, as the two switches both being on leads to a larger speed-up than each switch alone does.

**Control common effect and common cause question:**

Ted has built two robots with very special properties. When they see light that they like, it causes them to make music! The more light they see that they like, the more music they make! However, each robot likes different colors of light. When they see colors they don’t like, it causes them to make less music.

What color lights should Ted show the robots if he wants Robot A to make THE MOST music?

- A. Blue light
- B. Green light
- C. Red light and green light
- D. Red light and blue light

The correct answer is C since it maximizes music.

**Control causal chain question:**

Unlike humans, aliens can sometime plant thoughts into the minds of other aliens. That means if an alien thinks about a topic, like a certain food, it can make another alien think the same thought! Alien thought control isn’t perfect, though. A thought is transmitted 90% of the time. The other 10% of the time, nothing happens.

Imagine there are four aliens: Vop, Ker, Ogg, and Ant.

Now imagine you have a mind zapper that can put a thought in an alien’s head. If you want alien Ogg to think about ice cream, what is the BEST strategy?

- A. Zap Vop’s mind
- B. Zap Ker’s mind
- C. Zap Ant’s mind

This question is inspired by the stimulus of (Steyvers et al. 2003). The correct answer is B (Ker), as it is a direct cause of Ogg’s thoughts.

#### Procedure

Immediately after the instructions, participants rated their knowledge of diabetes management and personal finance.^11^ Participants answered two questions asking “Compared to other people your age, how would you rate your knowledge of diabetes management [personal finance]?” The answer choices were: I know a lot more, I know somewhat more, About the same, I know somewhat less, I know a lot less. We refer to this rating as participants’ perceived relative knowledge. Participants then completed the two real-world questions (in random order) and next the three control questions (in random order).^12^ In case any effects found in the previous experiments were driven by the set order of answer choices, we randomized the order of answers for all questions by participant. After the control questions, participants completed a set of questions designed to measure their actual knowledge in the finance and diabetes domains. We used the five multiple choice financial literacy questions developed by (Lusardi and Mitchell 2009; 2011) and a subset of the Diabetes Knowledge Questionnaire (DKQ), which contains a set of true/false questions. While the 24-item DKQ-24 (Garcia et al. 2001) is commonly used, we condensed it to eight questions to make the length more consistent with the financial literacy assessment. During the demographic questionnaire at the end of the study, we again asked the same question used in Experiments 2 and 3 to gather data on diabetes experience. We added two further questions to assess personal experience with personal finance. The questions were “Have you participated in (made active choices in) a retirement plan, such as a Roth IRA or 401K?” and “Have you made any investments like buying a CD (certificate of deposit), investing in stocks, or purchasing mutual funds?” with the response options being Yes, No, or Unsure. We refer to responses on these measures as personal domain experience.

### Results and discussion

**Fig. 7:**
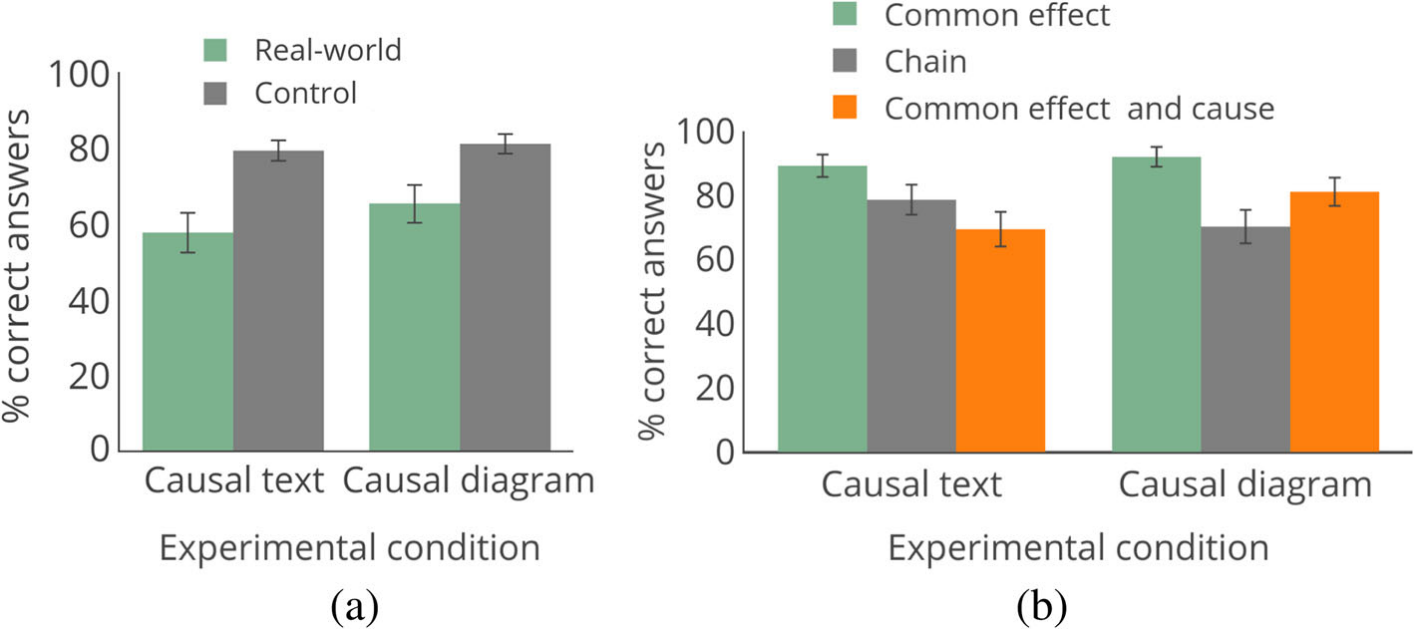
Experiment 4 results, focusing on control vs. real-world questions. Error bars show 95% confidence interval. **a** Comparing real-world and control questions. **b** Individual control questions

**Control vs. real-world decision-making.** In Experiments 1–3 our hypothesis was that participants had low accuracy specifically when they had domain experience, rather than because they are unable to use causal information. Our first analysis is thus comparing the performance (percentage correct responses) across the three control questions to the performance on the two real-world decision-making questions, irrespective of experience and knowledge. As shown in Fig. 7a, there is a significant difference in performance across the questions. Accuracy for control questions was 79.2% for the causal text versions and 81.0% for the causal diagram versions. On the other hand, accuracy for the real-world questions was 57.5% with causal text and 65.2% with diagram-based causal information. The performance difference between real-world and control questions was significant for both causal text and causal diagram versions (*p*s < 0.0001;*OR*=2.81 causal text, *OR*=2.28 causal diagram). This is consistent with our hypothesis that knowledge translation—rather than general unfamiliarity with or difficulty in causal thinking—is the root of the reduced performance in the earlier experiments.

Next, we examine results for each control question. As shown in Fig. 7b and Table 4 (which gives statistics for each answer choice), accuracy is highest for the simplest causal structure—the common effect—and there is no significant difference in accuracy between causal diagram or causal text versions (causal diagram = 92.0%, causal text = 89.2%, *p*=0.2472,*OR*=1.47). This shows that participants do understand causal information from both text and diagrams, and are able to use it to answer decision-making questions.

**Table 4:** Percentage of subjects selecting each answer for the control questions in Experiment 4

Common effect	A. Red	B. Green	**C. Red+green**	D. Blue
Causal text	4.1	5.4	89.2	1.4
Causal diagram	1.7	2.4	92.0	3.8
Common effect and common cause	A. Blue	B. Green	**C. Red+green**	D. Red+blue
Causal text	2.1	6.3	69.4	22.2
Causal diagram	3.0	5.0	81.1	11.0
Causal chain	A. Vop	**B. Ker**	C. Ant	
Causal text	16.6	78.6	4.8	
Causal diagram	23.4	70.2	6.4	

Correct answers are bolded, and answer order was randomized during study. For the causal chain question, answer A is the first node in the chain and a parent of the correct answer

We find that, with more complex structures, accuracy does decrease and information format may matter. First, the common effect and common cause question builds on the structure presented in the common effect question. Overall accuracy is lower than in the simpler case of the common effect (*p*<0.0001 collapsing across question formats). There is also a significant difference between causal text and causal diagram presentation of the information, with causal diagram accuracy being significantly higher (causal diagram = 81.1%, causal text = 69.4%, *p*=0.001,*OR*=1.89). Given the number of causal relationships described, the visual presentation may make it easier to see what information is relevant to the decision. Conversely, for the causal chain question, participants were more accurate with the causal text description than the causal diagram (causal text = 78.6%, causal diagram = 70.2%, *p*=0.0201,*OR*=1.56). The causal chain question has fewer nodes and edges than the other structures, which suggests that differences found with this structure are not due solely to complexity. Together, the results for the complex common effect and common cause and the simpler causal chain suggest that different information formats may potentially be more useful depending on the causal structure.

**Relationships between perceived relative knowledge, actual knowledge, and personal domain experience** Before examining the effect of individual factors on the use of causal information for decision-making, we first explore the relationship between perceived relative knowledge, actual knowledge, and personal domain experience. To measure actual finance knowledge, we use participants’ raw scores on the five questions of the finance literacy knowledge assessment. To measure actual diabetes knowledge, we use answers on the eight-question DKQ. There was a sparse tail in the distribution of responses to this question.^13^ As such, we group scores 0–3 into one measure. This leads to six groups (knowledge levels) for both questions.

**Fig. 8:**
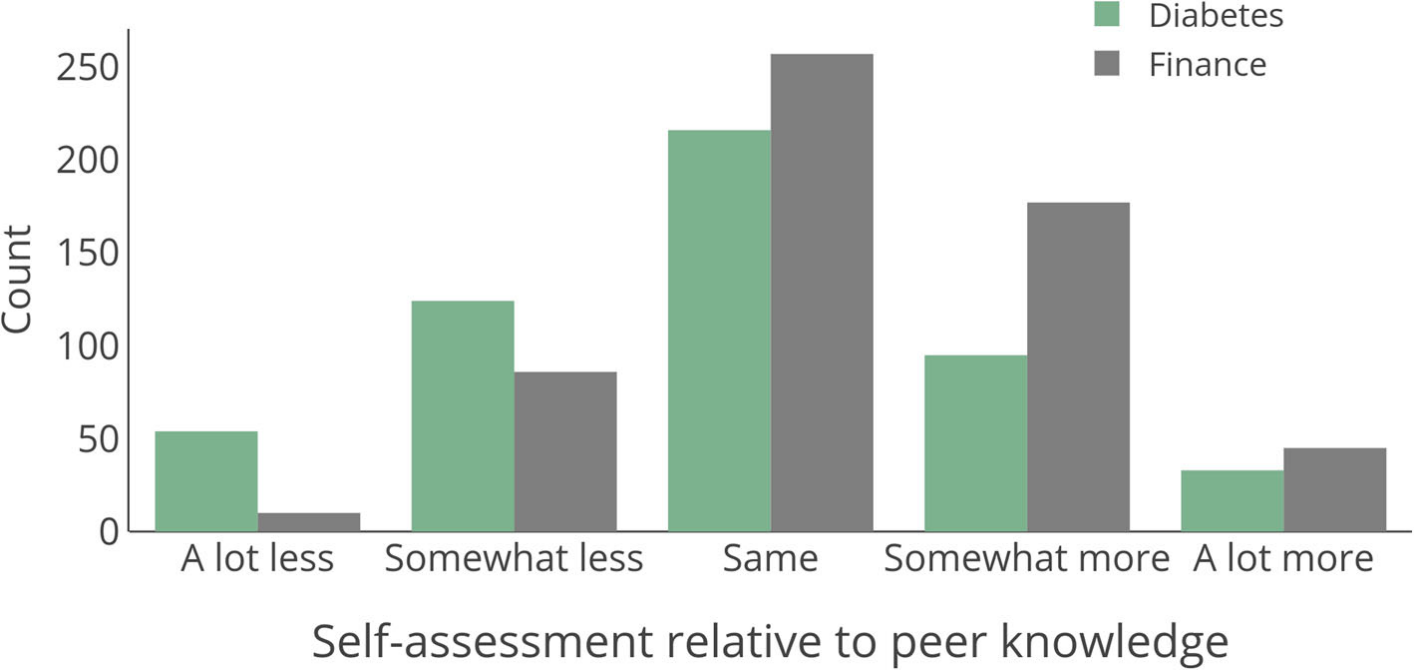
Self-assessment of knowledge in two domains

**Fig. 9:**
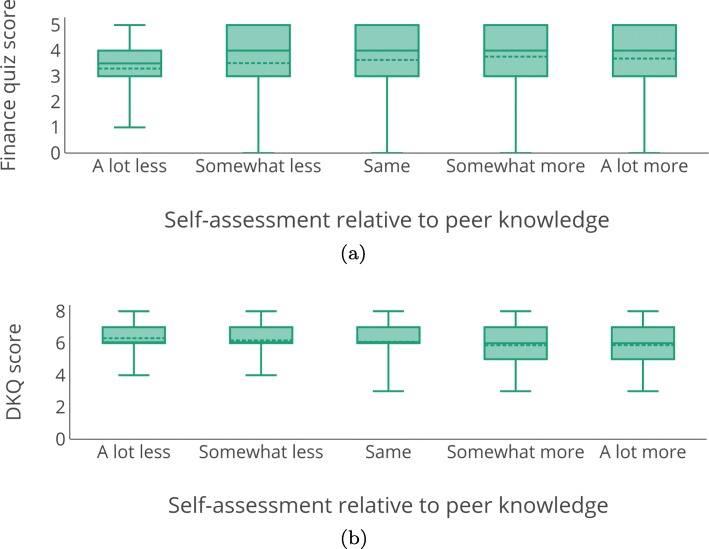
Self-assessed vs. actual knowledge for the two domains tested. Plots show median (*solid line*), mean (*dashed line*), and minimum/maximum. **a** Finance. **b** Diabetes

Figure 8 shows the distribution of self-assessed ratings of knowledge for each domain. Overall, participants were more confident in their knowledge of personal finance, with only 16.7% saying they know somewhat less or a lot less than others their age, while 34.1% provided that assessment of their knowledge of diabetes management (*p*<0.0001,*OR*=2.58). In both domains we find that participants’ self-assessments are not an accurate reflection of their domain knowledge. Figure 9a and b show the relationship between perceived and actual knowledge. Perceived and actual knowledge are somewhat negatively related in the diabetes domain (*ρ*=−0.088, *p*=0.044 using Spearman’s rank correlation), while they are unrelated in finance (*ρ*=0.065, *p*=0.118). That is, when individuals believed they had a high level of knowledge about diabetes management, they tended to do slightly worse on the knowledge assessment, while there was no relationship between people’s perception and their actual knowledge of personal finance. Thus, perception and actual knowledge are not redundant, and they measure different individual-level factors that may affect decision-making accuracy.

Perceived knowledge was, however, related to domain experience. For both domains participants were much more likely to rate themselves as having more knowledge when they had more experience in the domain (perceived knowledge and retirement experience: *ρ*=0.24, perceived knowledge and investment experience: *ρ*=0.35, perceived knowledge and diabetes status: *ρ*=0.23; *p*<0.001 for all correlations). Note that for the finance question, this was true regardless of whether we examine specifically retirement decision-making experience or investment experience more generally. Given that the two measures of finance experience behaved similarly, we conducted further analyses with just the retirement investment experience question, as it is more directly related to the decision-making question.

Actual knowledge did not show the same pattern of correlations with experience. For finance, a similar significant, positive correlation between personal domain experience and actual knowledge was found as with perceived knowledge (*ρ*=0.137; *p*=0.001). However, for diabetes, there was a significant, negative correlation between actual knowledge and experience (*ρ*=−0.127; *p*=0.004). Overall, these correlations suggest that people may be conflating experience with proficiency.

**Fig. 10:**
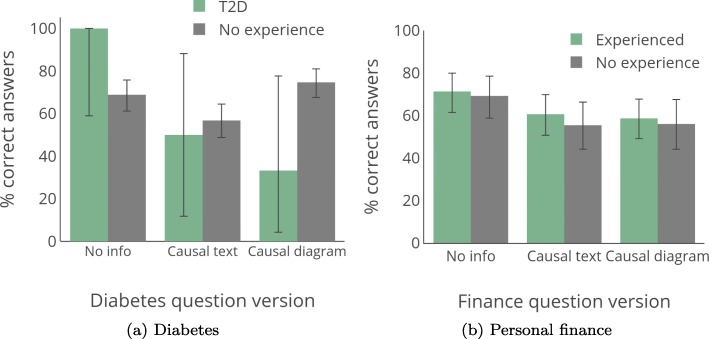
Results for real-world decision-making questions in Experiment 4 broken down by level of domain experience. Error bars show 95% confidence interval. **a** Diabetes. **b** Personal finance

**Effect of individual-level factors on decision-making accuracy** We now examine how individual-level factors affect decision accuracy and the use of causal information. To reduce the total number of comparisons, we collapse the causal diagram and causal text conditions to create a composite causal information condition.^14^ We present the results of the causal diagram and causal text condition separately in Table 5 and Fig. 10.

**Table 5:** Percentage of participants correctly answering the decision-making question for Experiment 4 by level of self-perceived expertise

Diabetes	Less	Same	More	Finance	Less	Same	More
No info	67.9	71.0	71.4	No info	80.6	68.9	66.7
Causal text	59.3	59.2	48.8	Causal text	58.6	58.0	58.5
Causal diagram	77.9	73.7	63.9	Causal diagram	51.6	55.8	62.2

We used an analytic approach that allowed us to test the impact on decision-making question accuracy of our manipulated variables of information condition and domain, while comparing across our collected variables measuring experience in these domains. Given that our domain variable was a repeated measure, we used a generalized estimating equations (GEE) approach. GEE allows for the same basic analytic approach as binary logistic regression, but it can handle repeated measures. Using a GEE analysis, the full model would include the three-way interaction of information condition x domain x experience. Examining the cross-tabulations for this interaction showed that 100% of the participants who had experience with diabetes in the no information condition correctly answered the decision-making question. In other words, there is a cell in our design that contains zero participants. This prevents us from including the three-way interaction involving experience. As such, we built a GEE model that included the variables of interest that could be included. We used a binary logistic link function to statistically model accuracy (incorrect vs. correct). We included the categorical variables of information condition (no info vs. causal information; between), domain (diabetes vs. finance; within), and personal domain experience (yes vs. no). We also included the continuous variables of relative knowledge and actual knowledge. To control for performance on the control questions, we included it as a predictor in the model. The full GEE model therefore included the three-way interactions of information condition x domain x perceived relative knowledge and information condition x domain x actual knowledge. We included the two-way interactions of information condition x domain, information condition x experience, domain x experience, information condition x perceived relative knowledge, information condition x actual knowledge, domain x perceived relative knowledge, and domain x actual knowledge. We also included the predictors of information condition, domain, experience, perceived relative knowledge, actual knowledge, and control question performance.

Using the model described above, we first examined whether there were any three-way interactions with domain, to inform whether to split by domain. While the information condition x domain x actual knowledge interaction was not significant, *p*=0.497, the three-way interaction of information condition, domain, and perceived relative knowledge was significant, *χ*^2^(1) =4.781, *p*=0.029. Given the significant three-way interaction and that, as described in the previous section, we found differences in how perceived knowledge was related to experience in the two domains, we split our analyses by domain to follow up on the interactions. Splitting the analyses by the only repeated-measures variable now provides for all between-subjects comparisons, allowing us to use the same logistic regression approach as in previous experiments.

We first examined accuracy for the finance domain. The finance question, where more participants reported knowledge of the domain, showed the same pattern of results as in Experiment 1, where we asked about another domain with which people were presumably familiar (weight management). Participants tended to perform worse with causal information as compared to no information, regardless of their experience or perceived knowledge. We conducted a two-step binary logistic regression with accuracy for the decision-making question (incorrect vs. correct) as our outcome. We entered information condition, experience, relative perceived knowledge, actual knowledge, and control question performance as variables in the first step of the model. We entered the interaction of information condition and experience, information condition and perceived knowledge, and information condition and actual knowledge in the second step. The final model was significant, *χ*^2^(8) =32.46, *p*<0.001, Nagelkerke *R*^2^=0.075. In Step 1, information condition was a significant predictor (*OR* =0.539, *p*=0.002), as was actual knowledge (*OR* =1.264, *p*=0.001). Control question performance was marginally significant (*OR* =1.231, *p*=0.085). No other predictors were significant, *p*s > 0.78. In Step 2, there were no significant interactions, *p*s > 0.15. We conducted a follow-up Fisher exact test to explore the significant information condition predictor. Accuracy with no extra information was 70.3%. This dropped to 58.0% with causal information (*p*=0.0256, *OR*=1.72).

We then examined accuracy for the diabetes domain. We conducted a two-step binary logistic regression with accuracy for the decision-making question (incorrect vs. correct) as our outcome. We entered information condition, experience, relative perceived knowledge, actual knowledge, and control question performance as variables in the first step of the model. We entered the information condition x perceived knowledge, and information condition x actual knowledge in the second step. Given the empty cell in our design, we could not enter the interaction of personal domain experience and information condition. The final model was marginally significant, *χ*^2^(7) =13.95, *p*=0.052, Nagelkerke *R*^2^=0.037. In Step 1, control question performance was significant (*OR* =1.398, *p*=0.013). No other predictors were significant, *p*s > 0.31. In Step 2, there were no significant interactions, *p*s > 0.13. Overall, this analysis suggests that for the variables we could include in our analysis, performance did not vary across these variables.

We could not include personal domain experience in the logistic regression analyses for diabetes because all of the T2D participants correctly answered the decision-making question in the no info condition. While we cannot conduct multivariate analyses that account for differences by personal domain experience, we can conduct exploratory analyses with Fisher exact tests to see if specific comparisons do differ by personal domain experience. These analyses should be seen as exploratory given the statistical issues noted. As shown in Fig. 10a, we show similar trends as in Experiments 2 and 3. The percentage of people without diabetes experience correctly answering the diabetes question does not differ across information conditions (no info = 68.9%, causal information = 66.1% accuracy, *p*=0.54744,*OR*=1.14). However, people with diabetes were more likely to do worse with causal information, dropping from 100% of people correctly answering the question in the no info condition to 41.7% of participants correctly answering the question with causal information (*p*=0.0174,*OR*=20.45). Note that in Experiments 2 and 3 we did not have a causal text condition for the diabetes-related question; there were only no info and causal diagram conditions. We combined the two causal formats for analysis for consistency, but briefly examine how the results here relate to the prior experiments. As can be seen in Fig. 10a, there are similar trends to Experiments 2 and 3, showing that individuals without diabetes tended to do better with a causal diagram than no info, though this was not statistically significant (increasing from 68.9 to 74.7% accuracy, *p*=0.2784,*OR*=1.34), while those with diabetes did worse (dropping from 100 to 33.3%, *p*=0.0210,*OR*=27.0). However, neither group performed better with causal text (T2D = 50.0% accuracy, no experience = 56.8% accuracy with text). In general across all experiments, results for causal text and causal diagrams were similar, so further work is needed to explore whether presentation format has different effects across domains.

In summary, Experiment 4 demonstrated that (1) participants are able to use causal information successfully with novel stimuli as seen in our control questions, (2) actual knowledge in a domain is not alone predictive of successful decisions with causal diagrams in that domain, (3) causal information can reduce accuracy when making decisions in familiar domains, and (4) while causal information either had no effect or reduced accuracy for those who had more experience, it can potentially assist those with less experience in some but not all domains.

## Discussion

### Combining causal information with prior knowledge

The key finding in this paper, demonstrated across multiple questions and domains, is that causal information at decision time can lead to less accurate choices in domains that relate to existing knowledge. Understanding causality conceptually (as in the control questions in Experiment 4 or prior work on learning (Rottman and Hastie 2016; Coenen et al. 2015; Rehder and Waldmann 2017; Gopnik and Sobel 2000)) does not necessarily imply being able to translate that knowledge to the types of decisions found in daily life.

We consider two potential explanations for our findings: ignoring new information and extra cognitive load. One might ask if the diagrams are helping individuals who are inexperienced (e.g., people without diabetes making decisions about T2D) because they actually use them, while people with experience assume they already have the necessary knowledge and ignore the new causal information. Ignoring information because it feels familiar would suggest a type of fluency effect (Oppenheimer 2008), where in familiar domains people may rely on quick System 1 thinking, and in less familiar domains may engage in System 2 deliberative thinking. Such a fluency explanation would predict that experienced participants are spending less time with the diagram than inexperienced participants. To test this possibility, we examined the time spent on decision questions as a function of personal experience in the relevant domain (i.e., T2D/no experience with diabetes, people who have made investments vs. those who have not) and question format (no info, causal diagram). We found no interaction between format and experience, and no effect of experience on time spent for any question.^15^ Since experience was not related to how long participants spent on the question, we do not believe differences in performance between individuals with and without domain experience are due to fluency effects causing experienced participants to ignore the causal diagrams. Further, if participants are simply ignoring the new information, then their performance should be similar to the no information conditions where the causal information was missing. Instead, across all experiments the information had either a positive effect (Experiments 2–4 for people without diabetes answering questions on diabetes management) or a negative effect (weight and personal finance questions).

Alternatively, it is possible that our results are related to the expertise reversal effect, whereby information may be redundant for experts and thus create extra cognitive load. Relevant to our study, one instance of the effect showed that adding causal words to Chinese texts aided novices but not students with higher reading proficiency (Kalyuga et al. 2013). It is possible that those who are proficient in a domain or have experience in it may have highly detailed mental models (Rouse and Morris 1986), which may not map well to the structure of our causal diagrams. That is, if an individual’s beliefs are at a different level of detail than that presented in the models, this may lead to conflicts when they see a simplified diagram or one that has significantly more detail than their own. This could explain why individuals do worse with new information than no information—if what they know is already correct, they can use it to answer questions, but the conflict with new information may have a detrimental effect. However, we do not believe this is the best explanation for our results. In Experiment 4 we found that while actual knowledge was not predictive of successful use of causal diagrams, it helped in answering the questions in general. On the other hand, experience did not lead to better results in either domain. Our findings suggest a key area for future work is to understand both the content of mental models and the confidence people have in them in the decision-making domains tested. That is, do people who do worse with causal information have more strongly held or detailed beliefs than those who do better? People who think they know a lot may have difficulty integrating the diagram with their beliefs (which may be stronger than the beliefs of people who do not think they have significant knowledge on the topic). More work is needed to investigate these mechanisms.

### Domain differences in decision-making

An interesting implication of our work is that there may be domain differences in how people use causal information for decision-making. We showed that causal information led to worse decisions about managing weight and personal finance. This was true regardless of experience level for the finance domain. In diabetes decision-making, however, people with no experience or low perceived knowledge did better with causal information. There may be aspects of prior experience relevant for decision-making that have not been captured in our experiments. In particular, people may be more likely to trust and use causal information in areas where they have not yet built their own intuitions (as in the control questions).

Similarly, it is possible that in some domains it is easier to understand how to apply causal information to a decision. An important difference between the diabetes and finance conditions is that, unlike retirement planning experience, diabetes experience is not voluntary. That is, if you are diagnosed with diabetes, there are things you have to do to maintain your health. Domains also differ in familiarity among laypeople. That is, one may read or hear more about how to manage one’s weight or finances as compared to how to manage or prevent diabetes. As a result, these domains may feel more familiar or self-relevant even if individuals do not have personal experience in them or are not successful at making decisions about them. Our data on perceived knowledge support this. We did not directly capture familiarity, but this may be related to the extent and strength of an individual’s beliefs about the domain. For example, participants may have more beliefs about personal finance than diabetes, which may lead to more challenges in using new causal information, due to conflict with existing beliefs. This may also be related to how personally meaningful people perceive the domain or decisions in it to be. All of these possible differences across domains are a fruitful ground for future exploration.

### Personalizing causal information

For individuals with some prior experience (and for all participants answering questions about financial decisions), simple causal models led to worse decisions and lower confidence than no information. This is a case where, based on prior work, there is a conflict between our understanding of causal learning and our understanding of decision-making. Work on cognitive load would suggest that the models should improve accuracy (Garcia-Retamero and Hoffrage 2006; Easterday et al. 2009), while work on judgment and decision-making has found that more information is not always helpful (Bastardi and Shafir 1998). Note that our results on confidence suggest there is more nuance in this assessment than suggested by prior work. For example, Fleisig (2011) found that individuals were more confident when given more information, even though performance was the same with and without the information. However, our results in Experiment 3 show that more information can in fact reduce confidence in decisions for individuals with experience in a domain. Reduced confidence may be appropriate if an individual is gaining appreciation for the complexity of a domain. Our task forced decisions, though, so it is possible that when there is an option to not act, this reduction in confidence may reduce the likelihood of making a choice entirely.

More work is needed to understand how to present only the relevant parts of a causal structure at the time of decision, while recognizing that what is relevant may differ across individuals and decision contexts. That is, not all variables relevant to a causal model are modifiable (e.g., family history), and others may be less desirable intervention targets for particular individuals (e.g., individuals who are more likely to stick to a diet program vs. an exercise regimen). Further, as we found in the control questions in Experiment 4, while causal diagrams may lead to higher accuracy with more complex structures, they may not be as effective as text for highlighting direct causes. Similarly, decision-making occurs under multiple constraints, including time. Thus, if some options are not applicable and a decision must be made quickly, a simplified version of the causal model may be more useful. On the other hand, it is possible that a structure that more directly links the causal information to specific actions may be better for some individuals. More work is needed on linking causal structures to people’s existing mental models, such as by eliciting information from users and developing diagrams based on that information. Since we believe that one difficulty for users is reconciling these two sets of information, it may be possible to develop computational approaches to assist in that process.

### Why keep humans in the loop?

One might wonder if the problem can be solved through automation. After all, computers can handle much higher levels of complexity, and they can make predictions using all available causal information. However, automation requires significant trust in a system, which is difficult to gain without first understanding it. Many high-stakes applications also cannot or should not be fully automated. For example, computational models have been used to estimate risk of recidivism to guide sentencing of defendants, but they may encode racial bias (Starr 2014). Even for behavioral nudges from wearable devices, users may be more likely to accept a prompt if they understand the immediate and long-term effects. When a suggestion seems incorrect or unusual, it is important for a human to be able to interrogate the AI system to find what the link is between their data, the suggestion provided, and their goals. Explainable AI and interpretable machine learning have focused on helping researchers understand how algorithms work (e.g., which visual features are being used for classification (Ribeiro et al. 2016)). A similar effort is now needed to help those who are not AI experts make sense of the output of algorithms and use them to guide decision-making.

### Limitations

While online experiments enabled a substantial sample size, one drawback is that we could not observe the decision-making process or conduct follow-up interviews with participants to probe how they used the information presented. Thus, a key limitation is that while we have hypotheses for the mechanism behind our findings, future work is needed to validate them. A second limitation is that our study focuses on individuals residing in the USA. Given what is known about cultural differences in causal judgments (Choi et al. 2003), we cannot say whether these results may apply to other populations. Additionally, we were not always able to collect large samples of participants with T2D. Future work could use methods that oversample this population to ensure larger sample sizes for experience groups of interest.

## Conclusions

Our ability to automatically extract causes from data is growing at a rapid pace, but little is known about whether causal information actually leads to better decisions in reality. Using large-scale online studies, we found that when people have some experience in a domain or believe they know about it, such as in the familiar domains of weight management and personal finance, causal information led to worse decisions with lower confidence. In contrast, individuals without diabetes-related experience made more accurate decisions in which they were more confident when given causal models. Future work is needed to better elicit an individual’s prior knowledge and to potentially personalize the information presented depending on their knowledge and the decision at hand. We found that text vs. graphical models representing causality can have different effects depending on the complexity of the model, suggesting a method is needed to quantifying the utility of a given causal representation.

## Appendix

### Demographics

Table 6 shows the demographics for the experiments.

**Table 6:** Demographics for all experiments

	Exp. 1	Exp. 2	Exp. 3	Exp. 4
Age range %				
18–24	10.1	9.1	14.7	10.8
25–34	46.3	44.3	46.7	49.1
35–44	26.2	27.5	22.3	25.3
45–54	11.7	12.0	10.6	8.9
55–64	5.6	7.0	5.7	5.8
Sex %				
Male	54.1	48.6	45.2	59.7
Female	45.9	51.4	54.8	40.1
Other	0	0	0	0.2
Ethnicity %				
Hispanic	8.4	6.9	7.3	10.2
Non-Hispanic	91.6	93.1	92.7	89.8
Race %				
American Indian or Alaska Native	1.9	1.4	1.6	0.5
Asian	8.0	6.2	8.2	7.3
Black or African American	9.4	8.2	9.0	10.8
Native Hawaiian/other Pacific Islander	0.3	0.5	0.1	0
White	78.0	83.2	77.8	75.8
Prefer not to say	0.5	0.3	0.6	0.5
Other	0.5	0.3	0.7	0.3

### Answer explanations

Here we provide more detail on the designated correct answers for each decision-making question.

#### Experiments 1 and 3 weight question

The correct answer to the question is B, as it is a direct cause of the effect. According to the diagram, answer C is an indirect cause of maintaining weight. Watching less TV does not map exactly to any of the nodes in the figure, and we do not know whether TV will be replaced by exercise or snacking. Finally, answer A is not as good as B, since A is below the recommended amount of physical activity shown in the diagram, whereas answer B fulfills the cause as shown. Further, calories can be consumed much faster than they are expended, so changes to diet can have a larger impact than changes to activity.

#### Experiments 2 and 3 diabetes question

In addition to answer C addressing two causes, answers A and B address only one component and D makes no change at all, making C the best answer, as it both reduces carbs and adds exercise.

#### Experiment 4 finance question

Option A has the potential for diversification, but stocks have higher volatility than cash, whereas answer B ensures (effectively) no potential loss of principal with no volatility. There is no mention of balancing growth in addition to reducing risk in the question, so B dominates A. This is because using the causal model, B fulfills two causes that lower risk (changing from a stock to cash introduces more diversity than simply switching between stocks). Answer C does not alter risk at all, while answer D strictly increases risk by exchanging an essentially risk-free asset for something that has more risk.

#### Experiment 4 diabetes question

While answer A may seem to relate to the family history component of risk, this choice cannot “undo” the existing family history, which is not a modifiable factor. Answer B could address weight, but it is not clear what type of food Alan will pick up, and this could easily increase his weight. Answer C strictly improves energy balance, since even if he consumes the same amount of soda, it will be accompanied by 30 min of walking each time. If Alan doesn’t walk, he will be drinking only water, reducing the amount of soda consumed. Finally, while reducing smoking (answer D) clearly addresses a cause of T2D risk, smoking status is a weaker risk factor than weight is (indicated by the plus signs in the figure and in the text), so this answer is not as good as C.

#### Experiment 4 common effect control question

Since the question states that Tim wants to make the conveyor belt go “as fast as possible,” there is only one right answer, as the speed will be lower if only the red switch or only the green switch is turned on. The blue switch has no effect on speed.

#### Experiment 4 common cause and common effect control question

Since Ted wants the robot to make the most music possible, including two causes is the best choice. This question has the same number of variables and the same structure as in question *common effect control question*, but now one of the causes of the common effect has a second effect (making it a common cause). The rationale for this question is to understand how much a small increase in complexity affects accuracy

#### Experiment 4 causal chain control question

The correct answer is the direct cause of the target alien’s thoughts. While A (Vop) could also be used to control Ogg’s thoughts, the question specifies that thoughts are not always transmitted, so a direct cause will be a better option than an indirect one. In pilot testing we used the text “Unlike humans, aliens can sometime plant thoughts into the minds of other aliens. That means, if an alien thinks about a topic, like a certain food, it can make another alien think the same thought! Alien thought control isn’t perfect, though, so the thought is not always transmitted.” However, at 75% the accuracy was lower than the other control questions, with the most common wrong answer being A (the first link in the causal chain). Thus, we made the probabilistic nature more obvious in the final version of the question. While we added probability information to this question after pilot testing to more clearly signal a preference for direct causes, this did not improve accuracy.

## Data Availability

The datasets used and/or analyzed during the current study are available from the corresponding author on reasonable request.

## Abbreviations

BG: Blood glucose
CBN: Causal Bayesian network
DKQ: Diabetes Knowledge Questionnaire
MTurk: Amazon Mechanical Turk
PWOD: People without diabetes
T1D: Type 1 diabetes
T2D: Type 2 diabetes

## Footnotes

1 For example, computer viruses (Lagnado and Sloman 2006), lightbulbs and amoebas (Rottman and Keil 2012), fictional aliens (Mayrhofer and Waldmann 2015), made-up diseases (Marsh and Ahn 2006), or “blicket detectors” (Gopnik and Sobel 2000).

2 See details from the National Weight Control Registry http://www.nwcr.ws/Research/default.htm, American College of Sports Medicine (Garber et al. 2011), and Obesity System Atlas (Vandenbroeck et al. 2007).

3 This question was used in Experiment 2 as well but was omitted in Experiments 3 and 4 to keep overall task durations similar.

4 In this and other figures error bars indicate 95% confidence interval, determined using the binomial exact calculation.

5 We explored whether the incorrect answers that were chosen differed by condition (see Table 1). Across all three conditions, “Go for a 30-min walk every weekend” was the most common wrong answer. The primary difference in results between causal text and the causal diagram came from twice as many participants selecting answer C (a statistically significant difference, *p*=0.0264), which could suggest a preference for root nodes in the causal diagram, as found in earlier work (Hagmayer and Sloman 2009; Hagmayer et al. 2007; Yopchick and Kim 2009; de Kwaadsteniet et al. 2010).

6 The “not helpful” group was much smaller, at 52 individuals.

7 For example, see the Joslin Diabetes Center’s guidance to patients: https://www.joslin.org/info/can_i_treat_diabetes_without_drugs.html.

8 To equate the amount of time spent on the experiment to Experiment 1 for payment purposes, we included the blicket question of Experiment 1 after the real-world question. This question always followed the real-world question for all participants. Because of the same issues described in Experiment 1, we do not analyze this question here.

9 We conducted a two-step logistic regression with the outcome of decision-making question accuracy (incorrect vs. correct) for just these two groups. We included personal domain experience (caregiver vs. PWOD) and information condition (no information vs. causal diagram) as variables in the first step of the model. The interaction of these two variables was entered in the second step. Personal domain experience was not a significant predictor in Step 1 of the model, *p*>0.4, and there was not a significant interaction in the second step, *p*=0.543. This suggests that caregivers and PWOD do not differ meaningfully in their responding.

10 For example, see Vanguard https://investor.vanguard.com/investing/how-to-invest/diversification, and (U.S. Securities and Exchange Commission 2010).

11 We ran another condition where participants did not rate their knowledge. Results were consistent with all main effects of interest, showing no significant result of the rating task.

12 We piloted the questions in a small-scale study with 108 individuals, primarily to evaluate whether highly detailed instructions on the meaning of causal diagrams would improve their use. There was no difference in accuracy between those who received detailed instructions and those who received the standard instructions (*p*=0.8992), so we used the same level of detail as in the other experiments so results would remain comparable. We made minor adjustments to language for clarity (e.g., referring to arrows instead of edges).

13 The counts of participants with scores [0,3] are 0, 4, 7, and 12 respectively, while 40 individuals scored a 4. On the other hand, 12 participants scored zero on the finance quiz.

14 We conducted the same generalized estimating equations analysis to explore decision-making question accuracy as used in the main analysis, but using only the causal diagram and causal text conditions, allowing us to statistically test whether these two conditions differed. There were no significant three-way interactions or two-way interactions, and the main effect of information condition was not significant, *p**s*>0.16. This analysis statistically supports collapsing these two conditions.

15 We analyzed total time spent on the page presenting the causal information and the decision options. For the diabetes decisions in Experiments 2 and 4 and the finance question of Experiment 4, we ran 2 (experience: T2D vs. no experience; finance experience vs. no experience) x 2 (question format: causal diagram vs. no info) analyses of variance (ANOVAs) on time spent answering the questions. For Experiment 2, we did not find a significant main effect of experience, *p*=0.847, or a significant interaction of experience and question format, *p*=0.965. For Experiment 4, we did not find a significant interaction for either decision question, *p**s*>0.86, or significant main effects of experience, *p**s*>0.89.

## References

[CR1] Balzer W.K., Sulsky L.M., Hammer L.B., Sumner K.E. (1992). Task information, cognitive information, or functional validity information: which components of cognitive feedback affect performance?. Organizational Behavior and Human Decision Processes.

[CR2] Bastardi A., Shafir E. (1998). On the pursuit and misuse of useless information. Journal of Personality and Social Psychology.

[CR3] Bui N., Yen J., Honavar V. (2016). Temporal causality analysis of sentiment change in a cancer survivor network. IEEE Transactions on Computational Social Systems.

[CR4] Busemeyer J.R., Townsend J.T. (1993). Decision field theory: A dynamic-cognitive approach to decision making in an uncertain environment. Psychological Review.

[CR5] Choi I., Dalal R., Kim-Prieto C., Park H. (2003). Culture and judgement of causal relevance. Journal of Personality and Social Psychology.

[CR6] Coenen A., Rehder B., Gureckis T.M. (2015). Strategies to intervene on causal systems are adaptively selected. Cognitive Psychology.

[CR7] Corter, J., Mason, D., Tversky, B., Nickerson, J. (2011). Identifying causal pathways with and without diagrams. In *Proceedings of the Cognitive Science Society*.

[CR8] Crump M.J., McDonnell J.V., Gureckis T.M. (2013). Evaluating Amazon’s Mechanical Turk as a tool for experimental behavioral research. PlOS One.

[CR9] de Kwaadsteniet L., Hagmayer Y., Krol N.P., Witteman C.L. (2010). Causal client models in selecting effective interventions: A cognitive mapping study. Psychological Assessment.

[CR10] Easterday M.W., Aleven V., Scheines R., Carver S.M. (2009). Constructing causal diagrams to learn deliberation. International Journal of Artificial Intelligence in Education.

[CR11] Fischhoff B., Davis A.L. (2014). Communicating scientific uncertainty. Proceedings of the National Academy of Sciences.

[CR12] Fischhoff B., Downs J. (1997). Accentuate the relevant. Psychological Science.

[CR13] Fleisig D. (2011). Adding information may increase overconfidence in accuracy of knowledge retrieval. Psychological Reports.

[CR14] Friedman, N., Murphy, K., Russell, S. (1998). Learning the structure of dynamic probabilistic networks. In *Proceedings of the 14th Conference on Uncertainty in Artificial Intelligence*.

[CR15] Friston K.J., Harrison L., Penny W. (2003). Dynamic causal modelling. Neuroimage.

[CR16] Fugelsang J.A., Thompson V.A. (2003). A dual-process model of belief and evidence interactions in causal reasoning. Memory & Cognition.

[CR17] Garber C.E., Blissmer B., Deschenes M.R., Franklin B.A., Lamonte M.J., Lee I.-M., Nieman D.C., Swain D.P. (2011). American College of Sports Medicine position stand. Quantity and quality of exercise for developing and maintaining cardiorespiratory, musculoskeletal, and neuromotor fitness in apparently healthy adults: guidance for prescribing exercise. Medicine and Science in Sports and Exercise.

[CR18] Garcia A.A., Kouzekanani K., Villagomez E.T., Hanis C.L., Brown S.A. (2001). The Starr County Diabetes Education Study. Diabetes Care.

[CR19] Garcia-Retamero R., Hoffrage U. (2006). How causal knowledge simplifies decision-making. Minds and Machines.

[CR20] Gopnik A., Sobel D.M. (2000). Detecting blickets: How young children use information about novel causal powers in categorization and induction. Child Development.

[CR21] Griffiths T.L., Sobel D.M., Tenenbaum J.B., Gopnik A. (2011). Bayes and blickets: Effects of knowledge on causal induction in children and adults. Cognitive Science.

[CR22] Griffiths T.L., Tenenbaum J.B. (2005). Structure and strength in causal induction. Cognitive Psychology.

[CR23] Hagmayer Y., Sloman S.A. (2009). Decision makers conceive of their choices as interventions. Journal of Experimental Psychology: General.

[CR24] Hagmayer, Y., Sloman, S.A., Lagnado, D.A., Waldmann, M.R. (2007). Causal reasoning through intervention. In: Gopnik, A., & Schulz, L.E. (Eds.) In *Causal learning: Psychology, philosophy, and computation*. Oxford University Press, Oxford, (pp. 86–100).

[CR25] Johnson D.R., Murphy M.P., Messer R.M. (2016). Reflecting on explanatory ability: A mechanism for detecting gaps in causal knowledge. Journal of Experimental Psychology: General.

[CR26] Kalyuga S., Ayres P., Chandler P., Sweller J. (2003). The expertise reversal effect. Educational Psychologist.

[CR27] Kalyuga S., Law Y.K., Lee C.H. (2013). Expertise reversal effect in reading Chinese texts with added causal words. Instructional Science.

[CR28] Karelaia N., Hogarth R.M. (2008). Determinants of linear judgment: A meta-analysis of lens model studies. Psychological Bulletin.

[CR29] Kim N.S., LoSavio S.T. (2009). Causal explanations affect judgments of the need for psychological treatment. Judgment and Decision Making.

[CR30] Kleinberg S. (2012). Causality, probability, and time.

[CR31] Kleinberg, S., & Elhadad, N. (2013). Lessons learned in replicating data-driven experiments in multiple medical systems and patient populations. In *AMIA Annual Symposium Proceedings*. PMC3900216 24551375

[CR32] Kominsky J.F., Zamm A.P., Keil F.C. (2018). Knowing when help is needed: A developing sense of causal complexity. Cognitive Science.

[CR33] Lagnado D.A., Sloman S.A. (2006). Time as a guide to cause. Journal of Experimental Psychology: Learning, Memory, and Cognition.

[CR34] Litman L., Robinson J., Abberbock T. (2016). Turkprime.com: A versatile crowdsourcing data acquisition platform for the behavioral sciences. Behavior Research Methods.

[CR35] Lu J., Xie X., Xu J. (2013). Desirability or feasibility: Self–other decision-making differences. Personality and Social Psychology Bulletin.

[CR36] Lusardi, A., & Mitchell, O.S. (2009). How ordinary consumers make complex economic decisions: Financial literacy and retirement readiness. *NBER Working Paper No. 15350*. https://www.nber.org/papers/w15350.

[CR37] Mitchell O.S., Lusardi, A. (2011). Financial literacy around the world: an overview. Journal of Pension Economics and Finance.

[CR38] Marsh J.K., Ahn W.-K. (2006). Order effects in contingency learning: The role of task complexity. Memory & Cognition.

[CR39] Mayer R.E. (2014). The Cambridge handbook of multimedia learning, 2nd edition.

[CR40] Mayrhofer R., Waldmann M.R. (2015). Agents and causes: Dispositional intuitions as a guide to causal structure. Cognitive Science.

[CR41] Oppenheimer D.M. (2008). The secret life of fluency. Trends in Cognitive Sciences.

[CR42] Pearl J. (2000). Causality: Models, reasoning, and inference.

[CR43] Polman E. (2012). Self–other decision making and loss aversion. Organizational Behavior and Human Decision Processes.

[CR44] Rehder B., Waldmann M.R. (2017). Failures of explaining away and screening off in described versus experienced causal learning scenarios. Memory & Cognition.

[CR45] Ribeiro, M.T., Singh, S., Guestrin, C. (2016). Why should i trust you?: Explaining the predictions of any classifier. In *Proceedings of the 22nd ACM SIGKDD International Conference on Knowledge Discovery and Data Mining*.

[CR46] Rottman B.M., Hastie R. (2014). Reasoning about causal relationships: Inferences on causal networks. Psychological Bulletin.

[CR47] Hastie R., Rottman, B. M. (2016). Do people reason rationally about causally related events? Markov violations, weak inferences, and failures of explaining away. Cognitive Psychology.

[CR48] Rottman B.M., Keil F.C. (2012). Causal structure learning over time: Observations and interventions. Cognitive Psychology.

[CR49] Rouse W.B., Morris N.M. (1986). On looking into the black box: Prospects and limits in the search for mental models. Psychological Bulletin.

[CR50] Rozenblit L., Keil F. (2002). The misunderstood limits of folk science: An illusion of explanatory depth. Cognitive Science.

[CR51] Shafir E., Simonson I., Tversky A. (1993). Reason-based choice. Cognition.

[CR52] Sloman S.A., Hagmayer Y. (2006). The causal psycho-logic of choice. Trends in Cognitive Sciences.

[CR53] Starr S.B. (2014). Evidence-based sentencing and the scientific rationalization of discrimination. Stanford Law Review.

[CR54] Steyvers M., Tenenbaum J.B., Wagenmakers E.-J., Blum B. (2003). Inferring causal networks from observations and interventions. Cognitive Science.

[CR55] Tenenbaum J.B., Kemp C., Griffiths T.L., Goodman N.D. (2011). How to grow a mind: Statistics, structure, and abstraction. Science.

[CR56] U.S. Securities and Exchange Commission (2010). Beginners’ guide to asset allocation, diversification, and rebalancing.

[CR57] Usher M., McClelland J.L. (2001). The time course of perceptual choice: the leaky, competing accumulator model. Psychological Review.

[CR58] Vandenbroeck P., Goossens J., Clemens M. (2007). Tackling obesities: Future choices. Obesity System Atlas.

[CR59] Woodward J. (2003). Making things happen: A theory of causal explanation.

[CR60] Yopchick J.E., Kim N.S. (2009). The influence of causal information on judgments of treatment efficacy. Memory & Cognition.

[CR61] Zeveney, A., & Marsh, J.K. (2016). The illusion of explanatory depth in a misunderstood field: The IOED in mental disorders. In: Pagafragou, A., Grodner, D., Mirman, D., Trueswell, J.C. (Eds.) In *Proceedings of the 38th Annual Conference of the Cognitive Science Society*. Cognitive Science Society, Austin.

